# Deep Learning Improves Pancreatic Cancer Diagnosis Using RNA-Based Variants

**DOI:** 10.3390/cancers13112654

**Published:** 2021-05-28

**Authors:** Ali Al-Fatlawi, Negin Malekian, Sebastián García, Andreas Henschel, Ilwook Kim, Andreas Dahl, Beatrix Jahnke, Peter Bailey, Sarah Naomi Bolz, Anna R. Poetsch, Sandra Mahler, Robert Grützmann, Christian Pilarsky, Michael Schroeder

**Affiliations:** 1 Biotechnology Center (BIOTEC), Center for Molecular and Cellular Bioengineering, Technische Universität Dresden, Tatzberg 47-49, 01307 Dresden, Germany; ali.al-fatlawi@tu-dresden.de (A.A.-F.); negin.malekian@tu-dresden.de (N.M.); ilwook.kim.1982@gmail.com (I.K.); sarah_naomi.bolz@tu-dresden.de (S.N.B.); anna.poetsch@tu-dresden.de (A.R.P.); 2 Department of Visceral, Thoracic and Vascular Surgery, University Hospital and Faculty of Medicine Carl Gustav Carus, Technische Universität Dresden, 01307 Dresden, Germany; sebastian.garcia@uniklinikum-dresden.de (S.G.); Beatrix.Jahnke@uniklinikum-dresden.de (B.J.); 3 Department of Electrical Engineering and Computer Science, Khalifa University of Science and Technology, Abu Dhabi 127788, United Arab Emirates; andreas.henschel@ku.ac.ae; 4 DRESDEN-Concept Genome Center, Center for Molecular and Cellular Bioengineering, Technische Universität Dresden, 01307 Dresden, Germany; andreas.dahl@tu-dresden.de; 5 Department of Surgical Research, Universitätsklinikum Erlangen, Maximiliansplatz 2, 91054 Erlangen, Germany; peter.bailey.2@glasgow.ac.uk (P.B.); Robert.Gruetzmann@uk-erlangen.de (R.G.); Christian.Pilarsky@uk-erlangen.de (C.P.); 6 National Center for Tumor Diseases (NCT), 01307 Dresden, Germany; 7 Department of Medical Oncology, Universitätsklinikum Dresden, 01307 Dresden, Germany; sandra.mahler@uniklinikum-dresden.de

**Keywords:** pancreatic cancer, chronic pancreatitis, transcriptome-wide association study, deep learning

## Abstract

**Simple Summary:**

Blood samples from patients with pancreatic diseases have been analysed to identify predictive RNA-based variants. These variants are not subject to changes in the environment, as is the case for gene expression or metabolic. The variants served together with CA19-9 as input to deep learning for a cohort of 268 patients with pancreatic diseases. Of these patients, 183 patients had pancreatic cancer and 85 from chronic pancreatitis. Among others, we were able to define a set of variants, which were able to differentiate resected pancreatic cancer from chronic pancreatitis with an area under the curve of (AUC) of 96%. Due to the ease of our approach and the wide availability of the used method, it will have a broad impact on the clinical routine. Suspicious patients are only subjected to a blood draw of 2.5 mL blood, and the specimen can then be sent at room temperature to a specialised laboratory.

**Abstract:**

For optimal pancreatic cancer treatment, early and accurate diagnosis is vital. Blood-derived biomarkers and genetic predispositions can contribute to early diagnosis, but they often have limited accuracy or applicability. Here, we seek to exploit the synergy between them by combining the biomarker CA19-9 with RNA-based variants. We use deep sequencing and deep learning to improve differentiating pancreatic cancer and chronic pancreatitis. We obtained samples of nucleated cells found in peripheral blood from 268 patients suffering from resectable, non-resectable pancreatic cancer, and chronic pancreatitis. We sequenced RNA with high coverage and obtained millions of variants. The high-quality variants served as input together with CA19-9 values to deep learning models. Our model achieved an area under the curve (AUC) of 96% in differentiating resectable cancer from pancreatitis using a test cohort. Moreover, we identified variants to estimate survival in resectable cancer. We show that the blood transcriptome harbours variants, which can substantially improve noninvasive clinical diagnosis.

## 1. Background

Pancreatic cancer is one of the deadliest diseases and the eighth most common cancer in Europe [1]. It accounts for 7–8% of cancer-related deaths in Europe, and the 5-year survival is less than 10% [2]. Two reasons for such poor prognosis are late diagnosis and misdiagnosis [3,4,5], as it shares many symptoms with chronic pancreatitis [6,7]. Late diagnosis can be ameliorated through non-invasive testing, which may lower the barrier for early monitoring. Diagnosis can be improved by an accurate differentiation between pancreatic cancer and chronic pancreatitis. Thus, it is vital to accurately stratify patients using noninvasive blood-based testing.

Currently, the most extensively evaluated biomarker for pancreatic cancer is a serum marker, Carbohydrate Antigen 19-9 (CA19-9) [8,9], an epitope of the sialylated Lewis blood group antigens [10]. In pancreatic cancer diagnosis, CA19-9 achieves a median sensitivity and specificity of around 80% [11]. There are two confounding factors for the limited validity of CA19-9 in early diagnosis: First, CA19-9 may be elevated in chronic pancreatitis and benign pancreatic tumours [12] and second, 5% of patients are Lewis a−/b− and cannot produce CA19-9 [13].

Recently, a number of blood-based approaches have been pursued to complement or go beyond CA19-9. Melo et al. described Glypican-1 as a cell surface marker for cancer exosomes [14]. Mayerle et al. took a different approach and analysed metabolic profiles to segregate pancreatic ductal adenocarcinoma (PDAC) and pancreatitis with an unsupervised machine learning model using principal component analysis (PCA) [7]. Mellby et al. established a signature of 29 biomarkers obtained from an antibody microarray for the same task [15]. Their results were promising, but the variability of metabolic concentrations and expression levels pose a challenge.

A complementary line of thought investigates genetic predisposition. Various authors have proposed mutations associated with pancreatic cancer. Childs et al. introduced four [16] and Grant et al. eleven mutations [17] associated with pancreatic cancer. However, Grant et al. concluded that the proportion of affected patients was small (3.8%), limiting the approach’s applicability.

Milne et al. and Klein et al. agreed in the argument that due to the complexity of the genetic component of pancreatic cancer, single variants of high penetrance genes like *BRCA1/2, INK4A, STK11, Tp53, APC,* and *ATM* could not fully explain the pathogenesis [18,19]. Variants of low penetrance genes might contribute to the susceptibility of the patient cohort because of a lack of immunosurveillance [20]. Growing evidence suggests that changes in immune cell composition and tumour microenvironment are associated with tumour progression [21,22].

By and large, many biomarkers achieve good classification results but with high variability, while known genetic variants are invariable predispositions but with limited applicability. Here, we seek to overcome these two limitations by combining them. We build on CA19-9 as an established biomarker and add high-quality and significant variants in expressed genes obtained from blood samples.

**Figure 1:**
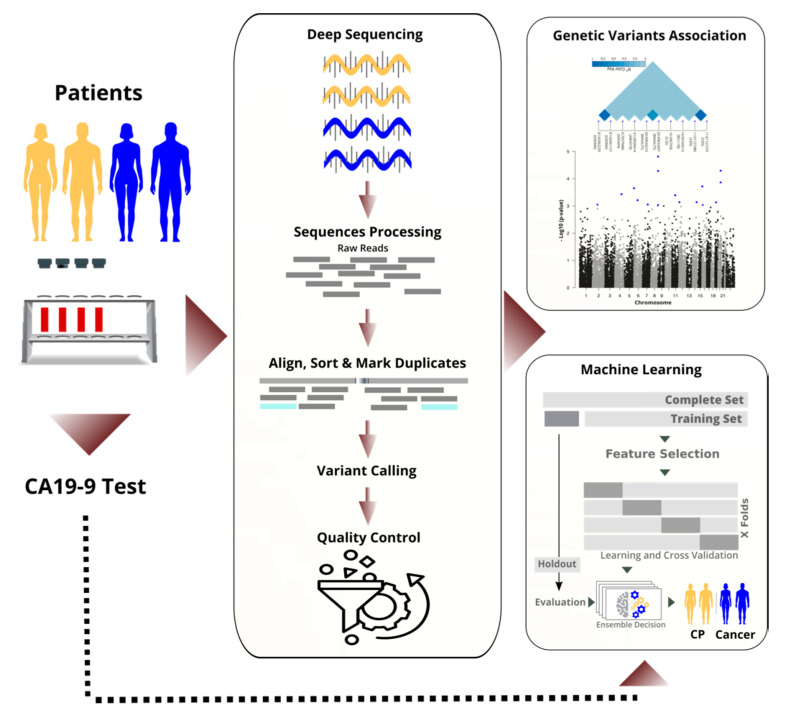
Blood samples were sequenced and processed. Millions of raw transcriptomic variants reduced to hundreds of high-quality, significant ones. Variants, together with CA19-9, were used to stratify patient groups. In the machine learning workflow, the dataset was divided into training and test sets before feature selection and optimisation. The test set was held out for evaluation only.

We sequenced the RNA of nucleated cells found in peripheral blood and achieved high quality through high coverage in sequencing and rigorous filtering of variants. Next, we performed a transcriptome-wide association study to identify statistically significant and predictive RNA-based variants (see Figure 1). By performing the analysis on the RNA instead of the DNA, we focused on important variants that are in the regulatory or coding region, and we saved costs. We discuss selected variants and their known links to cancer biology. 

In parallel to the TWAS, we applied machine learning to differentiate cancer from chronic pancreatitis (CP), and in particular, resectable PDAC (rPDAC) from CP. Finally, we demonstrate that our approach is even capable of defining a signature to estimate survival for resectable PDAC.

## 2. Material and Methods

### 2.1. Local Ethics Committee

Patients with histopathologically confirmed resectable pancreatic ductal adenocarcinoma (*n* = 87, rPDAC), non-resectable pancreatic cancer (*n* = 96, nrPC), and confirmed chronic pancreatitis (*n* = 85, CP) were included in the study. Cancer patients were classified by clinicians as non-resectable either because they were distant metastatic or locally advanced. The study was reviewed by the internal review board of the University Hospital Dresden, Germany. After approval from the local ethics committee at the University Hospital Dresden (reference number: *EK349122008*), all patients gave their written informed consent to take part in the study.

### 2.2. Sample Preparation and Deep Sequencing

Venous blood samples were extracted from the patients using the PAXgene Blood RNA Tube System (PreAnalytiX, Hilden, Germany) before surgery or any other planned medical intervention. Until further analysis, the samples were stored at −20 °C. CA19-9 was determined in serum using CLIA (DiaSorin). RNA was isolated from the blood using the PAXgene Blood RNA Kit (PreAnalytiX, Hilden, Germany) according to the manufacturers’ instruction. Contaminating genomic DNA in the RNA samples was removed by the treatment of the RNAs with Baseline Zero- DNase (Epicentre, Madison, WI, USA). After the clean-up of the digestion with RNeasy Mini Kit (Qiagen, Hilden, Germany), the RNA samples were subjected to quality control using the Agilent RNA 6000 Nano Kit (Agilent, Waldbronn, Germany). Only samples displaying a RIN > 8.0 were subjected to RNA sequencing. Messenger RNA was isolated from 1.5 µg total RNA by combining the depletion of haemoglobin using the Globin-Zero Gold Kit (Epicentre, Madison, WI, USA) with successive poly-dT enrichment using the NEBNext Poly-A mRNA Magnetic Isolation Module according to the manufacturers’ instruction. Final elution was done in 15 µL 2× NEBnext first-strand cDNA synthesis buffer (NEB, Frankfurt, Germany). 

After chemical fragmentation, by incubating at 94 °C for 15 min, the samples were directly subjected to the workflow for strand-specific RNA-Seq library preparation (Ultra Directional RNA Library Prep, NEB, Frankfurt, Germany). For ligation, custom adaptors were used: Adaptor-Oligo-1: 5′-ACACTCTTTCCCTACACGACGCTCTTCCGATCT-3′, 2: 5′-PGATCGGAAGAGCACACGTCTGAACTCCAGTCAC3′.

Indexing was performed during the following PCR enrichment (15 cycles) using custom amplification primers carrying the index sequence indicated with “*NNNNNN*” (Primer1: Oligo_Seq5′ AATGATACGGCGACCACCGAGATCTACACTCTTTCCCTACACGACGCTCTTCCGATCT-3′, Primer2: 5′ CAAGCAGAAGACGGCATACGAGAT-NNNNNN-GTGACTGGAGTTCAGACGTGTGCTCTTCCGATCT-3′).

For Illumina sequencing, samples were equimolarly pooled and distributed on the respective number of flow-cells for 75bp single-end sequencing on Illumina HiSeq 2500, resulting in an average of 60 Mio fragments sequences per sample.

### 2.3. Sequence Assembly and Quality Control

FASTQC 0.10.1, Cutadapt 1.9.1, and STAR 2.7 were used sequentially for quality checking, adapters removal, and mapping. Sequences were aligned and annotated based on Genome Reference Consortium Human Build 38 (GRCh38). To estimate the complexity and predict the redundancy of a genomic sequencing library at a given sequencing depth, we used two software packages: Preseq and Genomecov (bedtools 2.30). We analysed sequences depth and coverage based on the aligned bam files. Picard 2.9 was used to mark PCR duplicates and sort reads. Reads were split by N operators of the CIGAR strings into component reads and trimmed into splice junctions to remove RNA overhangs by using the Genome Analysis Toolkit (GATK 4.1 and GATK 3.8). GATK’s base quality score recalibration (BQSR) estimated possible systematic sequencing errors [23]. The HaplotypeCaller of GATK single-nucleotide polymorphisms (SNVs) and insertion-deletions (INDELs) simultaneously via the local de novo assembly of haplotypes in an active region [23]. Then these variants were hierarchically merged over the samples into one file in GVCF format and then subjected to hard filters. Hard-filtering throws out variants below specific thresholds for properties, such as variant confidence, root mean square of the mapping quality and strand bias. Other standard quality control protocols were applied [24,25] by using PLINK 2. We adopted very stringent criteria to ensure the quality of the study and validity of the results of statistical analysis results. Missingness filter, for example, excludes a massive number of variants that were present in less than 97% of our samples. The second important filtering measure was the minor allele frequency (MAF). MAF is the rate of occurrence for the second most common allele in the given population.

For samples, the following quality control steps were applied: missingness, which excludes the samples with high missing variants; heterozygosity, which filters samples with too high or low heterozygosity rate, relatedness, which find pairs of samples looking too similar to each other, and finally stratification, which checks whether the samples belong to the same population. Table S1_1 gives an overview of the tools and configurations described in this section.

### 2.4. Statistical Analysis

The transcriptome-wide association study (TWAS) was implemented as a logistic regression model on the 16,934 high-quality variants using PLINK 2. Three cohorts of patients were used for different analysis, namely rPDACr, nrPC, and CP. The significant variants that have a *p*-value with minus four orders of magnitude or better were studied. We also evaluated rare variants that passed the minor allele frequency (MAF) filter and showed a distinct level of association (e.g., with an odds ratio of around ten). We still call them rare, although they pass the MAF filter because of their relatively low MAF (around 10%). In all of our statistical analysis, gender was added as a covariate in the logistic regression models to prevent bias. However, this has an ignorable influence on the variants ranking.

### 2.5. Feature Selection for Machine Learning

Firstly, the dataset was divided into a training set (80%) and a test set (20%). The feature selection process was performed with a logistic regression model on the 16,934 high-quality variants using only the training set. Variants were ranked and evaluated independently for each task (cancer vs. CP, rPDAC vs. CP, nrPC vs. CP). By using the training set, the features (variants) were ranked according to their significance (*p*-value and odds ratio). We applied principal component analysis on an incremental number of variants to identify a minimum number of features that present a higher possible segregation level. For each task, variants with a *p*-value below 0.005 or an odds ratio higher than ten (or below 0.1) were nominated and kept as features. They were normalised to a binary format (zero for the reference allele and one for an alternative allele). CA19-9 was used as a feature and fed to the models in a binary format (below or higher than the clinical threshold of 37 U/mL).

### 2.6. Machine Learning

A deep feed-forward neural network approach was implemented and optimised with Adam optimiser in a fivefold cross-validation process on the training set. Networks were built for the tasks and optimised with Keras 2.2.4. It consisted of a visible layer, five dense fully connected hidden layers with ReLu activation functions, and an output layer with Softmax function. A batch normalisation and dropout of 0.4 were applied for some tasks. For stochastic learning, the Adam optimiser [26] was used with learning rate decay. The fivefold cross-validation process resulted in five models, which were optimised and evaluated separately (see Figure 1). At the final evaluation, the test set (a holdout) was used. For each task, the area under the ROC curve (AUC), accuracy, precision, sensitivity, and F1-score were calculated and reported. We calculated the average performance of the five models. Additionally, we ensembled decisions of the five models per task by averaging their probabilities in identifying the positive class. Overall, the two averaging methods gave very close measures in all classification tasks.

### 2.7. Survival Analysis

A linear regression, using *PLINK 2,* was applied to the training set to evaluate the predictivity of each variant on its own. Box-Cox power transformation was applied to approximate the normal distribution of the survival time [27] of rPDAC samples. Then, Kaplan–Meier plots and log-rank test were performed to evaluate the best variant.

The multivariate analysis, using a proportional-hazards model (Cox regression), was implemented to define a signature of multiple variants by using the R packages: *survminer* and *survival*. The backward elimination selection procedure [28] on the training set kept the effective predictors as features and excluded detrimental or time-dependent ones. The likelihood ratio test, Wald test, and the log-rank test were applied.

To check if the requirements for the proportional hazards model are met, the proportional hazards assumption of the Cox regression was tested by using the Schoenfeld residuals against the transformed time. Having high *p*-values (e.g., above 0.05) in this test indicates that there are not time-dependent coefficients in the final model; therefore, the assumptions are valid. By using the test set, the time-dependent area under the ROC curve [29] and the covariate specific ROC curve [30] were determined.

## 3. Results

In this study, we aimed to explore combining an established biomarker with RNA- based variants to accurately distinguish pancreatic cancer in its two profiles (resectable and non-resectable) from chronic pancreatitis, as well as to estimate survival in the resectable type. Of the sequenced patients, 68% suffered from pancreatic cancer and 32% from chronic pancreatitis. Most of the cancer patients were above 60 years old, while most pancreatitis patients were between 50 and 60 years old. The cancer group was gender-balanced, while the pancreatitis group was predominantly male. Gender was included as a covariate to accommodate the imbalanced regression models. CA19-9 values were obtained for nearly all cancer and pancreatitis patients. Survival for rPDAC was nearly twice as long as for non-resectable cancer (see Table 1).

In clinical practice, a CA19-9 value higher than 37 U/mL is an indication of pancreatic cancer [9]. With around 84% AUC and 76% accuracy, CA19-9 distinguishes cancer from pancreatitis and serves as a baseline. Adjusting the clinical threshold for CA19-9 does not improve classification results because false positives and false negatives do not cluster around the threshold of 37 U/mL, but they spread out widely (see Figure S1_1).

Overall, we rigorously reduced millions of raw variants to hundreds of high-quality, highly significant variants. Our transcriptomic-wide association study identified variants highly associated with pancreatic cancer or chronic pancreatitis. Encouraged by these results, we trained machine learning models and tested them using an independent test set. The receiver operating curves demonstrate: Variants on their own differentiate the resectable pancreatic cancer from chronic pancreatitis with 89% AUC. Combined with CA19-9, they reach 96%. Deep learning on high-quality, highly significant variants together with CA19-9 nearly perfectly distinguishes resectable PDAC from pancreatitis using 76 RNA-based variants.

### 3.1. Sequencing at 60 Mio Read Depth

We sequenced the RNA of blood samples at 60 Mio read depth. We examined the utility of further sequencing and optimisation of the sequencing depth. Figure S1_2 shows the average of all reads in each base position over our samples. Some of the base positions were covered with less than ten reads, which are likely to be lost if we sequence with less coverage. Importantly, the significant variants that were selected as features for machine learning (i.e., with a *p*-value below 0.005) are in the middle region of the graph, where base positions were covered with at least 50 reads. This means that it is possible to reduce the cost and to sequence at a lower depth without losing significant information. For example, sequencing with 30 Mio instead of 60 Mio fragments will result in keeping the hypergeometric probability of having more than 20 reads in each of these positions around 95% (by assuming that calling variants requires around 20 reads). 

Furthermore, we computed the expected yield of distinct fragments of reads for experiments smaller than the input experiment (c_curve) by resampling our bam files for all samples. We found that sequencing with an average of 30 Mio fragments per sample instead of 60 Mio will not lead to losing more than 0.6 Mio distinct fragments out of 7 Mio, which corresponds to around 9% (see Figure S1_3).

### 3.2. Consistency of Cell Types between Groups

**Figure 2:**
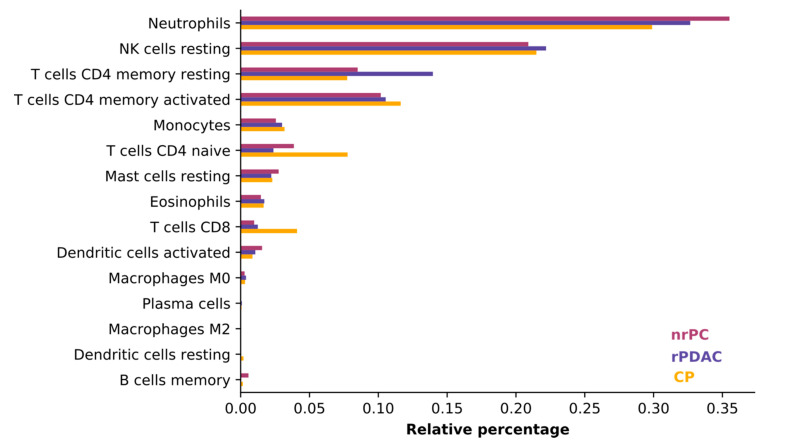
CIBERSORT results for the four groups of patients. Neutrophils, NK, and T cells are the most abundant cell types across all groups.

We judged the average consistency of cell types between groups using CIBERSORT, which estimates the abundances of member cell types in a mixed cell population, using gene expression data [31]. All blood samples were comprised mostly of neutrophils, natural killer cells (NK cells), and T cells. The composition of cells was consistent and did only differ in the least abundant cell types (see Figure 2) so that the difference in cell type composition between patient groups does not influence variant calling.

### 3.3. Variants and Samples Quality Control

Our sequences have a high base call accuracy with Phred quality scores per base pair position above 25 (see Figure S1_4). To obtain high-quality variants, we followed standard quality control procedures [24,25]. In the quality control workflow, 2,039,151 raw variants were reduced to 16,934 high-quality variants in around 9000 genes. Mostly, variants were filtered out because of their high missingness among samples or their low minor allele frequency. Missingness initially excluded 93% of variants. After exclusion of variants with low genotype quality, another 28% of the variants were removed. Minor allele frequency filtering removed 80% of variants. All other steps had a comparatively minor impact. The sample’s quality control removed around 5% (see Table 2).

### 3.4. Statistical Analysis (TWAS)

From the high-quality variants, we identified highly significant variants through carrying out a transcriptome-wide association study. First, we sought variants differentiating pancreatic cancer in its two profiles (rPDAC or nrPC) from chronic pancreatitis (CP). Then we compared each profile with CP (see Figure S1_5 for Manhattan plots). Table 3 shows the identified significant variants for each analysis. Overall, four variants in the genes *GSDMD, B4GALT5*, and *VPS36* were significantly associated with pancreatic cancer, with *p*-values of minus four orders of magnitude or better. Other variants were associated with CP with high affinity. Moreover, six variants present in 10% or below of our samples were highly significant. More than 90% of these variants appeared in cancer, and only less than 10% were in CP. One of them (rs3093553) was already defined as a risk factor for breast cancer in two GWAS studies (see Table 4).

An analysis for linkage disequilibrium showed that alleles occur together less often than expected on the same haplotype (negative linkage disequilibrium) in the two variants of *GSDMD*. For the two variants of *CD5*, two alleles occur together more often than expected on the same haplotype (positive linkage disequilibrium). The two variants in *B4GALT5* were in moderate positive linkage disequilibrium. Variants in different genes were independent (see Figure S1_5 for linkage disequilibrium plots).

### 3.5. Deep Learning Accurately Differentiates Cancer

By using logistic regression on the training set, we carried out a feature ranking and selection process. We selected variants with a *p*-value below 0.005 or an odds ratio higher than 10 (or below 0.1) as features for our machine learning model. The numbers of the obtained features were 70, 76, and 67 for cancer vs. CP, rPDAC vs. CP, and nrPC vs. CP, respectively (see Sheet S2_1, Sheet S2_2 and Sheet S2_3 for the full lists). Overall, we rigorously reduced millions of variants to hundreds of high-quality, highly significant features. We do not only exploit affinity towards cancer but also against cancer (associated with CP). Every single variant achieved an AUC lower than 67% on its own, which is below the predictivity of CA19-9. 

**Figure 3:**
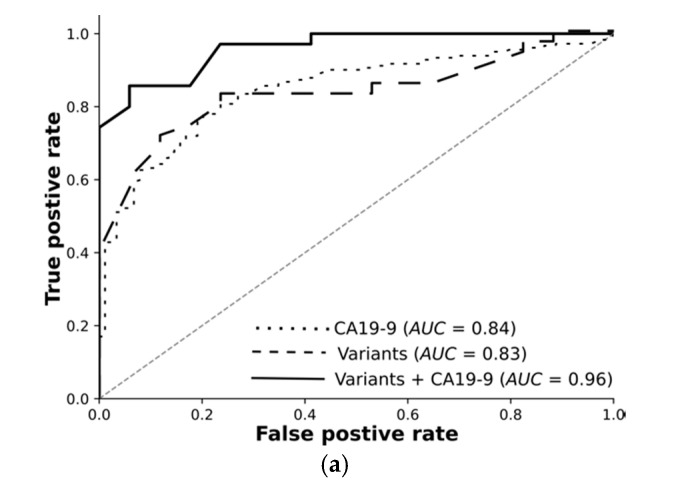

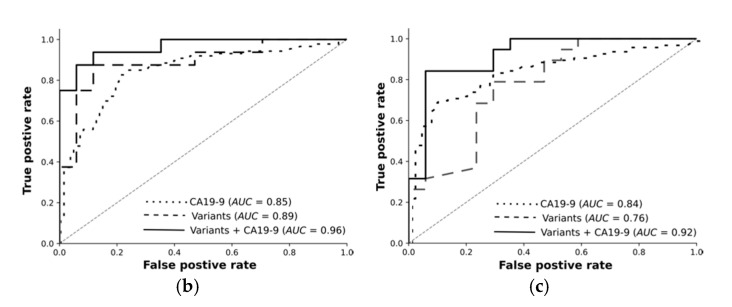
Receiver operating characteristic for analyses shown in Table 5. (**a**) Cancer vs. CP, (**b**) rPDAC vs. CP, and (**c**) nrPC vs. CP.

For each classification task, we trained feed-forward deep neural networks by using the selected features and another by using the selected features and CA19-9 together. The models were optimised using the training set with fivefold cross-validation and evaluated using an independent test set. The receiver operating curves in Figure 3 demonstrate: Deep learning on high-quality, highly significant features (variants) together with CA19-9 nearly perfectly distinguishes resectable PDAC from pancreatitis and improves the performance of CA19-9 on its own from 84% to 96%. Our models significantly improve the diagnosis across all classification tasks. The selected features without CA19-9 achieved an AUC of 89% in differentiating rPDAC from CP. Table 5 and Figure 3 summarise AUC as well as accuracy, precision, and recall for all tasks and show the predictivity of variants and CA19-9 separately and together. Performance of each fold in the cross validation on training and test sets are reported in Table S1_2 and Table S1_3, respectively. 

### 3.6. Estimating Survival

Next, we estimated survival from variants. In our patient cohort, resectable PDAC patients survive nearly twice as long as non-resectable ones (see Table 1). In the group of resectable PDAC, we applied a linear regression model for evaluating the association between each variant on its own with survival time in a univariate model. Among the 16,934 high-quality variants, *rs6728689* in *SP100* was highly associated with poor prognosis in rPDAC. 

**Figure 4:**
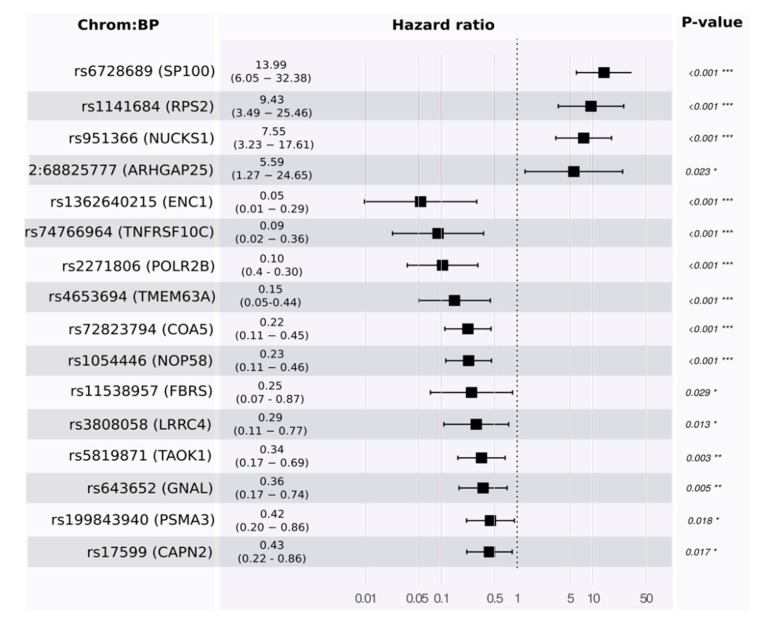
Forest plot for the proportional hazard model to visualize the size effect of the 16 significant variants in estimating survivals in rPDAC. The asterisks symbols next *p*-values are used to highlight the significance; the more asterisks, the higher the significance. Global *p*-value (Log-Rank) = 2.284 × 10^−22^; Concordance = 0.89.

**Figure 5:**
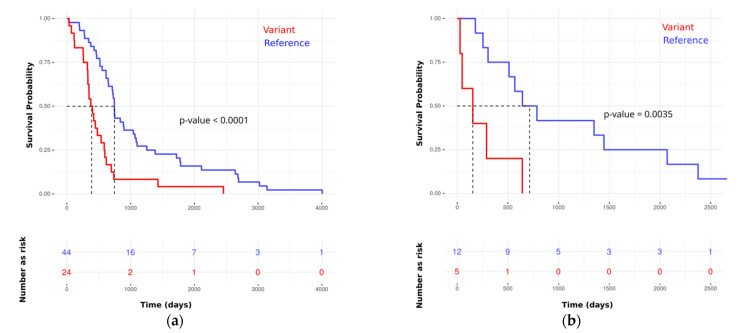
Kaplan–Meier plots for best variants (rs6728689 in SP100) show a high association with poor prognosis in rPDAC. The y-axis represents survival probability, and the x-axis represents the survival time in days. The vertical dotted lines represent the median of survival (survival probability of 0.5) in each group. (**a**) Using the training set; (**b**) using the independent test set.

Toward a multivariate model, we developed a proportional hazards model to estimate survival. Using the training set, our model identified 16 features. Some of these were associated with good prognosis and others with poor prognosis (see Figure 4 and Table S1_4). *p*-values for the likelihood ratio test, Wald test, and the log-rank test were below $1\times 10^{-9}$, and the concordance was 0.89 (standard error = 0.19). The main driver in the model was rs6728689 (in SP100), which shows a distinctive level of significance with a *p*-value of $6.87\times 10^{-10}$ and a hazard ratio of around 14. Evaluating its predictivity using the independent test set reveals a high level of association (see Figure 5 for Kaplan–Meier plots in the training and testing sets).

Checking for proportionality assumption by using the Schoenfeld residuals against the transformed time showed that the global model and each of the selected predictors were having *p*-values above 0.05. In this test, having very small *p*-values is an indication of time-dependent coefficients, which is not desirable; however, that was not the case in our analysis (see Table S1_5). Therefore, the requirements for the proportional hazards model are met, and the hazard rate of an individual is relatively constant in time. Using the test set, we computed time-dependent AUC and the covariate specific AUC, and they were 92% and 89%, respectively.

### 3.7. Variants’ Biological Connection to the Disease

The variants were obtained from peripheral blood, but interestingly, the genes harbouring the variants appear to play a role in cancer tissue. Table 3 summarises the significant variants. In the analysis of cancer (rPDAC + nrPC) vs. CP, three variants in the genes *GSDMD* and *B4GALT5* showed a high association with pancreatic cancer in its two profiles (rPDAC and nrPC). In the gene *GSDMD*, one variant was significantly associated with cancer and another with CP. Wang et al. discuss *GSDMD’s* potential as a cancer target due to its role in pyroptosis gasdermin mediated programmed cell death [32]. It is highly connected with the CASP gene family (see Figure S1_6a), which is strongly related to the poor prognosis of pancreatic cancer [33].

Interestingly, studying other possible associations in the data showed that the variant of *B4GALT5* was associated with patients that were false negatives of CA19-9. This observation is in agreement with Indellicato et al. in pointing out an association between *B4GALT5* and glycosylation profiles and its connection to the elevation of CA19-9 values [34]. Generally, *B4GALT5* is a membrane-bound glycoprotein, which is associated with *MUC4* (see Figure S1_6b, STRINGDB [35]). *MUC4* is associated with pancreatic cancer and was proposed as a marker to differentiate pancreatic cancer from pancreatitis [36]. 

Other variants were identified because of their significant association with CP. Some of them were already studied and defined in other genomic association studies as markers for other diseases such as type 2 diabetes (see Table 3).

In the survival analysis, *rs6728689* in *SP100* was highly associated with poor prognosis in rPDAC. *SP100* can activate p53-dependent transcription, which is important for regulating apoptosis by supporting the stimulatory effect of homeodomain interacting protein kinase-2 (*HIPK2*) [37]. Accordingly, we suggest that variations in *SP100* may reduce the activity of *HIPK2* and attenuate p53-dependent apoptosis.

## 4. Discussion

In 1981, Koprowski et al. introduced CA19-9 based on a small case study with two pancreatic cancer patients [38]. Today, it serves as a monitoring tool after pancreatic cancer resection. Its use in diagnosis is still limited because of its low accuracy of around 80% [11]. To date, there is no single biomarker or genetic variant that outperforms CA19-9 in detecting pancreatic cancer. However, combinations of biomarkers or variants [7,15,18,19] promise improvements. 

In our approach, we wanted to exploit the synergy between CA19-9 as an established clinical biomarker with RNA-based variants, which capture a form of dynamic predisposition obtained from the transcriptome. Combining the tumour marker CA19-9 with signatures of selected variants in a deep learning approach results in highly accurate classification. They span a variety of tasks, such as distinguishing cancer from CP and estimating survival in the resectable type. Interestingly, deep learning with variants as input but without CA19-9 achieves a performance comparable to the established CA19-9 on its own. This means that our approach can replace CA19-9 for Lewis-antigen negative patients, where CA19-9 on its own is not applicable.

In differentiating rPDAC from pancreatitis, we obtained an AUC of 96%, which is above the 94% AUC reported in [7] and significantly above the 84% AUC in [15]. But importantly, their approaches were built on biomarkers, which are continuous, namely metabolic and gene expressions, respectively. They could carry noise inherent to the method and read-out. In contrast, we observe discrete features whose read-out is binary (present or absent) and whose noise can be controlled by the sequencing depth. Thus, we believe that our selected features provide a very robust and stable signature, which can be analysed in laboratories specialised in genetic analysis without the need for special equipment. Moreover, the accuracy (or F1 score) of our models indicates that we were able to improve the diagnosis by 14% over CA19-9, whose accuracy is 77%.

In fact, we believe that many of the selected variants can contribute to a causal disease model. In agreement with Milne et al. and Klein et al., we do not solely focus on well-known high penetrance genes but include low penetrance genes [18,19]. We found that many of the identified variants do not directly affect protein function and structure, as they are located in the three prime untranslated regions (*3′-UTR*). These variants can influence tumour susceptibility by polyadenylation, translation efficiency, localisation, and stability of the mRNA [39].

Although some of the genes harbouring variants appear to play a role in cancer tissue, it was not our primary intention to find genes expressed in the tumour tissue or, for that matter, to find mutations. For example, speaking about tumour tissue, one would expect to capture signals from KRAS mutations, especially in non-resectable cancer (nrPC), which includes metastasis patients. Nevertheless, there is no single variant in KRAS that shows any level of significance for all the comparison groups. Therefore, the assay was developed mainly to perform diagnosis without tumour tissue to support the performance of the biomarker CA19-9 and introduce a reliable diagnosis model. The basic idea started from attempts to evaluate gene expression changes in the nucleotide cells of peripheral blood, but it successfully resulted in developing a marker panel in single-nucleotide variants (germline variants) because of its superior accuracy and higher stability.

The germline variants do not change over time and the only dynamic component in the developed model is CA19-9, but it is not fully precise to argue that we introduced a patient sub-stratification system based on static predispositions. Rather, we successfully exploit the synergy between the dynamic biomarker CA19-9 and those static predisposition variants. Moreover, there is another dynamic component in the model, which is the expression of the genes carrying the variants. While the variant itself is static, its gene is behaving dynamically and may or may not be expressed. We consider only variants on expressed genes, and hence it is a combination of static and dynamic aspects.

Some variants may be pinpointed due to indirect correlation with other phenomena associated with the false diagnosis of CA19-9. For example, variants in the gene *B4GALT5* were associated with rPDAC in our data. Evaluating all possible associations indicates that these two variants were also associated with false negatives of CA19-9 diagnosis, and this was exploited by our model to improve CA19-9 prediction. This was not a surprise due to several studies linking *B4GALT5* to glycosylation and then to the elevation of CA19-9 values [33]. Generally, *B4GALT5* is a membrane-bound glycoprotein, and it was linked with pancreatic cancer through *MUC4* [36,40]. Cataloguing germline predispositions is invaluable for cancer screening, prevention, and early detection. However, this does not necessarily mean that PDAC or CP is encoded in the patients’ DNA. The patient’s dynamic response to the disease may have a static variant signature that we utilised in our prediction. 

Furthermore, we exploit affinity towards cancer and also against cancer (associated with CP). We believe that the former can also be used to indicate pancreatic cancer compared with normal patients without pancreatitis. Some of the cancer variants were particularly associated with rPDAC or nrPC, and others with pancreatic cancer in general (see Table 3).

Finally, a key to these results is high-quality data. We sequenced at 60 Mio coverage of high-quality mRNA. While whole-exome sequencing (WES) is primarily for DNA variant discovery and RNA-Seq is mainly for measuring gene expression, both can be used to detect single nucleotide variants (SNVs) [41]. WES is the sequencing of genomic DNA that has been enriched for exonic regions. This is cheaper than sequencing an entire genome. RNA-seq is even cheaper and less complicated than WES. By performing the analysis on the RNA level instead of the WES, we focused on important variants that are in the regulatory or coding region, and we saved costs. Furthermore, as shown in Figure S1_3, half the coverage leads to a 9% decrease, while double the coverage to a 10% increase. In comparison to the reduction of variants due to missingness, these changes are small, and a further increase in coverage is unlikely to have a positive effect. However, the large reduction due to missingness in some samples means that future improvements in sequence quality are likely to increase the number of significant variants, which is unlikely to improve the nearly perfect classification results but likely to improve a causal understanding of the disease processes.

## 5. Conclusions

We have shown that deep sequencing and machine learning can significantly improve pancreatic cancer diagnosis from blood samples. The combination of the established biomarker CA19-9 with new high quality and significant RNA-based variants resulted in the ability to differentiate pancreatic cancer from pancreatitis with an AUC of 96%. We also defined a signature of 16 variants significant for estimating survival in resectable PDAC.

Conceptually, our approach combines dynamic and static read-outs. CA19-9 captures a dynamic reaction to cancer, while the variants in the expressed genes are static predisposition. The former may reflect dynamic disease progression, while the latter captures dynamic predispositions obtained from the transcriptome. As a first step towards a deeper understanding of the causal relationships, we discussed the variants that were significant for cancer. In particular, the genes *B4GALT5* and *GSDMD*, which harbour three of the most significant variants, are closely related to cancer progression and the elevation of CA19-9 levels. In an additional analysis, we identified six statistically significant variants, one of which was already defined as a breast cancer risk factor in several publications. However, these six variants are having limited impact due to their lower minor allele frequency (around ten).

Overall, our results show that deep sequencing and machine learning can pave the way to early and accurate diagnosis as well as personalised treatment options.

## Figures and Tables

**Table 1:** Patients’ age, gender, survival, and CA19-9 values.

Category	Cohort rPDAC (*n* = 87)	Cohort nrPC (*n* = 96)	Cohort CP (*n* = 85)
	*n* (%)	*n* (%)	*n* (%)
**Gender**			
Male	46 (53%)	51 (53)	73 (86%)
Female	41 (47%)	45 (47%)	12 (14%)
**Age (years)**			
<50	8 (10%)	8 (8%)	36 (42%)
50–60	10 (11%)	19 (20%)	35 (41%)
<60	69 (79%)	69 (72%)	14 (17%)
Median	69	68	51
Q1, Q3, IQR	62, 74, 12	60, 73, 13	45, 56, 11
**Median of survival (days)**	615	297	NA
**CA 19-9 Values**			
≤37	24 (28%)	27 (28%)	68 (80%)
>37	61 (70%)	68 (71%)	16 (19%)
Unknown	2 (2%)	1 (1%)	1 (1%)

**Table 2:** Quality control (QC) steps for variants and samples. The workflow resulted in 16,934 high-quality variants for 255 high-quality samples.

Type	QC Step	Passed *n* (%)	Excluded *n* (%)
**Variant QC**		2,309,151	
	1. Hard filters	2,028,582 (87.8%)	280,569 (12.2%)
	2. Missingness	124,078 (7%)	1,904,504 (93%)
	3. Genotype quality (GQ) filter	89,614 (72.3%)	34,464 (27.7%)
	4. Minor allele frequency (MAF)	17,961 (20%)	71,923 (80%)
	5. Hardy Weinberg Equilibrium	16,934 (95.7%)	757 (4.3%)
		16,934	
**Samples QC**		268	
	6. Missingness	268 (>99%)	1 (<1%) {1 nrPC}
	7. Heterozygosity	265 (99%)	3 (1%) {1 nrPC, 1 rPDAC and 1 CP}
	8. Relatedness	260 (98%)	5 (2%) {3 nrPC, and 2 CP}
	9. Stratification	255 (98%)	5 (2%) {2 nrPC, 1 rPDAC and 2 CP}
		255	

**Table 3:** The significant variants (with a *p*-value of minus four orders of magnitude or better) for different analysis on our data. The last column (additional association) gives information about more associations for the variant in our data or citations for that variant in other GWAS studies (PMIDs are provided). OR is odds ratio.

SNV BP	Rs Id	Gene	*p*-Value	OR	Additional Association
**Cancer (rPDAC + nrPC) vs. CP:**					
8:143561151	rs12541790	GSDMD	$1.54\times 10^{-5}$	3.26	In particular, with nrPC
20:49633437	rs11471493	B4GALT5	$5.11\times 10^{-5}$	2.60	rPDAC & CA19 False Negative (*p*-value = $2.81\times 10^{-4}$)
8:143562235	rs11551198	GSDMD	$5.21\times 10^{-5}$	0.31	
20:49634210	rs235032	B4GALT5	$1.37\times 10^{-4}$	2.17	rPDAC & CA19 False Negative (*p*-value = $1.44\times 10^{-4}$)
16:3067936	rs2239303	IL32	$1.91\times 10^{-4}$	0.43	Another GWAS study with acute lung, PMID: 21649914
4:128039414	rs11098945	ABHD18	$3.72\times 10^{-4}$	0.47	Other GWAS studies with type 2 diabetes,
					PMIDs: 26964836, 25774817, 25145545, 24843659 & 21490949
11:61127379	rs375347163	CD5	$4.04\times 10^{-4}$	0.35	
11:61127380	rs72912997	CD5	$4.04\times 10^{-4}$	0.35	
6:70579486	rs1048886	SDHAF4	$6.16\times 10^{-4}$	0.43	Other GWAS studies with type 2 diabetes,
					PMIDs: 26964836, 25774817, 25145545, 24843659 & 21490949
14:99169513	rs375191905	BCL11B	$7.27\times 10^{-4}$	0.36	
**rPDAC vs. CP**					
12:9083754	rs1385820032	A2M	$5.59\times 10^{-5}$	0.22	
16:3067936	rs2239303	IL32	$7.04\times 10^{-5}$	0.34	
8:23188960	rs1334003684	RP11-1149O23.2	$8.37\times 10^{-5}$	0.24	
15:61856531	15:61856531	RP11-16B9.1	$8.80\times 10^{-5}$	0.3	
14:99169513	rs375191905	BCL11B	$1.38\times 10^{-4}$	0.23	
20:49633437	rs1555810424	B4GALT5	$2.11\times 10^{-4}$	2.86	CA19 False Negative (*p*-value = $1.2\times 10^{-4}$)
20:49634210	rs235032	B4GALT5	$2.24\times 10^{-4}$	2.49	CA19 False Negative (*p*-value = $1.2\times 10^{-4}$)
3:64000218	rs112759850	PSMD6-AS2	$2.94\times 10^{-4}$	0.11	
12:107733077	rs1045749	PRDM4	$4.45\times 10^{-4}$	0.42	
3:70957989	rs1387507994	FOXP1	$4.48\times 10^{-4}$	0.27	
13:52413342	rs13431	VPS36	$6.70\times 10^{-4}$	2.36	Another GWAS study with Ulcerative colitis, PMID: 27902482
12:107733387	12:107733387	PRDM4	$8.41\times 10^{-4}$	0.43	
12:115821	rs66898998	MED13L	$9.33\times 10^{-4}$	0.43	
17:42315197	rs368910594	STAT3	$9.54\times 10^{-4}$	0.33	
17:50862895	17:50862895	TOB1	$9.86\times 10^{-4}$	0.13	
4:128039414	rs11098945	ABHD18	$9.92\times 10^{-4}$	0.43	
**nrPC vs. CP**					
8:143561151	rs12541790	GSDMD	$8.93\times 10^{-5}$	3.541	
8:133458886	rs879077709	ST3GAL1	$9.08\times 10^{-5}$	0.21	
8:143562235	rs11551198	GSDMD	$1.50\times 10^{-4}$	0.26	
2:74969604	rs7881	POLE4	$2.11\times 10^{-4}$	0.347	
2:96834655	rs10643982	CNNM3	$2.71\times 10^{-4}$	0.33	
6:70579486	rs1048886	SDHAF4	$3.72\times 10^{-4}$	0.3	
8:17229247	rs1043093	CNOT7	$7.59\times 10^{-4}$	0.12	
17:1736164	rs11549259	WDR81	$9.30\times 10^{-4}$	0.41	
**rPDAC vs. nrPC**					
12:131922463	rs3088051	ULK1	$6.31\times 10^{-5}$	0.34	Another GWAS study with Crohn’s disease, PMID: 22536218
12:27755670	rs3751233	MRPS35	$7.91\times 10^{-4}$	0.30	
11:112084759	rs544184	C11orf57	$8.01\times 10^{-4}$	2.17	Another GWAS study with colorectal cancer, PMID: 26377099

**Table 4:** Significant variants with low minor allele frequency were additionally identified, mainly in cancer patients. The last three columns show the number of the samples with these variants in all groups, only in cancer groups, and only in CP. Number in cancer and CP in this table means number of samples that carry this variant.

SNV BP	Rs Id	Gene	No. in Cancer *n*	No. in CP *n*	Additional Association
11:77616654		CLNS1A	29	3	
11:77616653		CLNS1A	29	3	
6:31581779	rs3093553	LTB	23	4	Another GWAS study with breast cancer, PMID: 23095343 & 21523452
5:140535437		ANKHD1	23	3	
7:36707016		AOAH	22	2	
9:34634803		SIGMAR1	21	1	

**Table 5:** Classification results of CA19-9 only, variants (with deep learning), and CA19-9 and variants together (with deep learning). Variants together with CA19-9 achieved very high performance, especially for rPDAC vs. CP.

Task	Variable		Performance on the Test Set				
	Type	No of Features	AUC	Precision	Recall	F1	Accuracy
Cancer vs. CP	CA19-9	1	0.84	0.89	0.72	0.79	0.75
	Variants	70	0.83	0.76	0.83	0.79	0.71
	CA19-9 & Variants	71	0.96	0.89	0.97	0.93	0.90
rPDAC vs. CP	CA19-9	1	0.85	0.78	0.72	0.75	0.77
	Variants	76	0.89	0.88	0.88	0.88	0.88
	CA19-9 & Variants	77	0.96	0.93	0.88	0.90	0.91
nrPC vs. CP	CA19-9	1	0.84	0.80	0.72	0.76	0.76
	Variants	67	0.76	0.68	0.89	0.77	0.72
	CA19-9 & Variants	68	0.92	0.75	0.95	0.84	0.81

## Data Availability

Sequence data in BAM format is available on the European Genome phenome Archive www.ebi.ac.uk/ega, accessed on 20 April 2021 and the EGA ID for the study is EGAD00001006915.

## Supplementary Materials

The following are available online at https://www.mdpi.com/article/10.3390/cancers13112654/s1, Figure S1_1: CA19-9 values of the target classes. Figure S1_2: (a) Manhattan plot shows the mean of the total reads for each base position over samples. (b) A chart to summarise the coverage of the distinct reads that are pointed in (a). Figure S1_3: Sequencing depth analysis using population sampling models to infer the behaviour under less or more in-depth sampling. (a) Median of the c_curves over the samples for the expected complexity. (b) Median of lc_extrap_curves over the samples, which simulates the expected future yield of distinct fragments of reads bound by the number of total distinct fragments in the library (lc_extrap_curves) based on bootstrapping when sequencing deeper. Figure S1_4: Sequences quality. (a) Phred quality scores per base pair position, (b) Phred quality scores per read. Figure S1_5: Manhattan plots to show the p-value of the evaluated variants (on the left side), and heatmap for linkage disequilibrium for the loci of the significant variants, which are identified in Table 2. (a) and (b) for cancer vs CP analysis. (c) and (d) for rPDAC vs CP analysis. (e) and (f) for nrPC vs CP. Figure S1_6: Protein-protein interactions of the genes: B4GALT5 and GSDMD from STRING Database. Table S1_1: An overview of the functions and configurations for variants calling and quality control using GATK & Plink. Table S1_2. Performance of each fold in the cross-validation on training and test sets for deep learning model using variants and CA19-9 together. Table S1_3. Performance of each fold in the cross-validation on training and test sets for deep learning model using variants only. Table S1_4. The significant predictors of the proportional hazard model for estimating the survival time of rPDAC patients. Table S1_5. Results of checking for proportionality assumption by using the Schoenfeld residuals against the transformed time. Sheet S2_1, Sheet S2_2 and Sheet S2_3 includes lists for the description for the selected features (variants) that have been used in the machine learning.
