# Supplementary material for: Deep Learning Improves Pancreatic Cancer Diagnosis Using RNA-Based Variants
Source: Cancers (Basel). 2021 May 28;13(11):2654. doi: 10.3390/cancers13112654 (PMC8199344; doi:10.3390/cancers13112654)
Supplement: Supplementary file 1 [file cancers-13-02654-s001.zip › Supplementary_S1.pdf]

cancers

MDPI

Supplementary S1

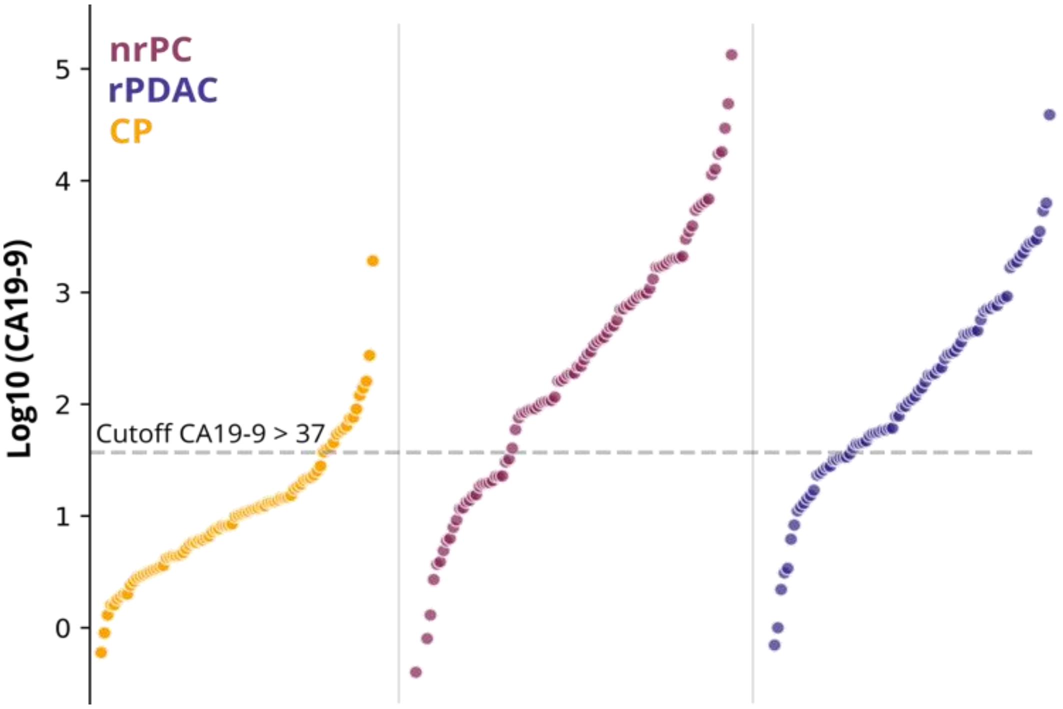

Figure S1_1. CA19-9 values of the target classes. The y-axis shows the CA19-9 values for the samples sorted by a[illegible] grouped by classes (84 CP samples in orange, 95 nrPC samples in red, and 85 rPDAC samples in Navy Blue). The dash[illegible] line shows the clinical cut-off (37 U/ml). Overall, 67 samples are misclassified.

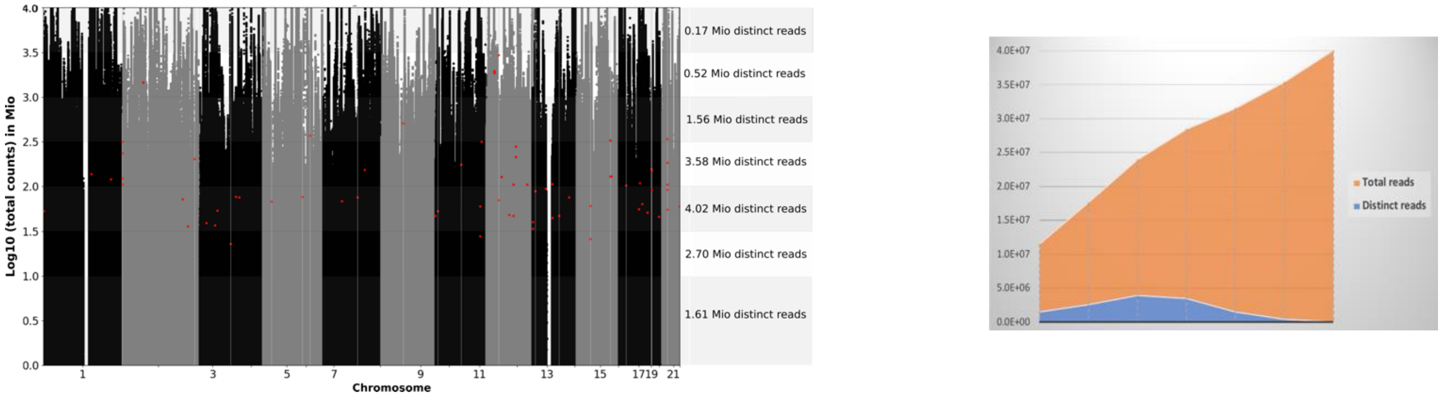

Figure S1_2. (a) Manhattan plot shows the mean of the total reads for each base position over samples. The x-axis represents th[illegible] chromosomes and their base positions, and the y-axis shows the number of the total reads for each of these positions. The red dot[illegible] represent variants with a given level of significance (p-value below 0.005) for rPDAC vs CP. It is clear from the figure that the significan[illegible] variants are in the middle region, and they are covered with 25 to 1000 reads. (b) A chart to summarise the coverage of the distinct read[illegible] that are pointed in (a).

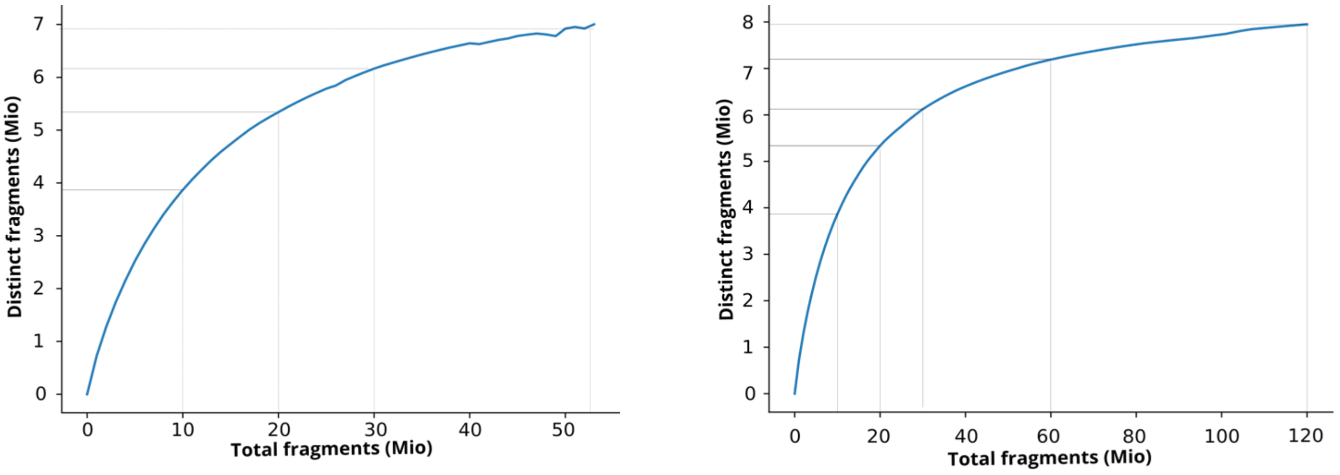

(a) *(b)*

Figure S1_3. Sequencing depth analysis using population sampling models to infer the behaviour under less or more in-depth sampling. (a) Median of the c_curves over the samples for the expected complexity. The x-axis gives the total number of fragments, and the y-axis shows the corresponding number of distinct fragments. (b) Median of lc_extrap_curves over the samples, which simulates the expected future yield of distinct fragments of reads bound by the number of total distinct fragments in the library (lc_extrap_curves) based on bootstrapping when sequencing deeper. The x-axis gives the total number of fragments, and the y-axis shows the corresponding average expected number of the distinct fragments. Sequencing with an average of 120 Mio fragments per sample instead of 60 Mio will lead to a gain of less than 0.8 Mio distinct fragments out of 8 Mio, which corresponds to around 10%.

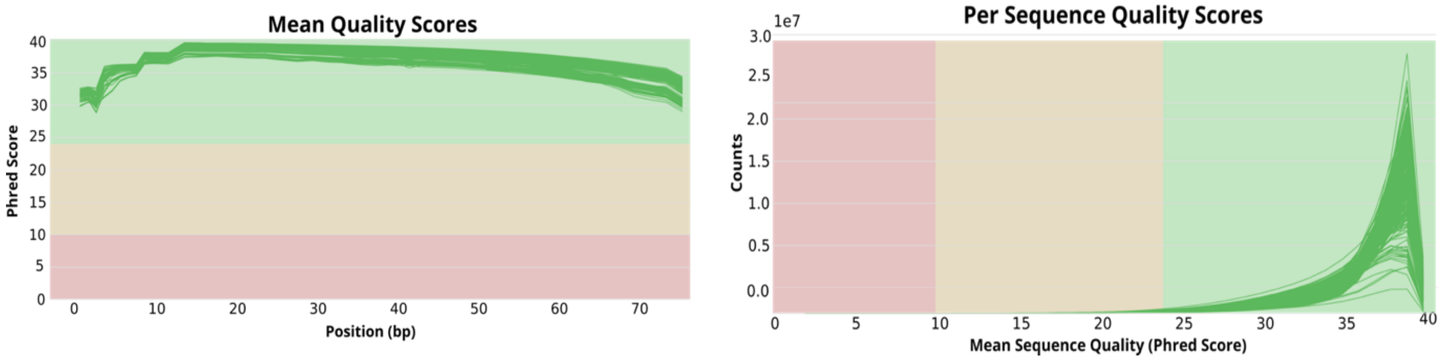

(a) *(b)*

Figure S1_4. Sequences quality. (a) Phred quality scores per base pair position, (b) Phred quality scores per read. Our sequences have a high base call accuracy with Phred quality scores per base pair position above 25, which means that the chances of incorrectly calling for that base are below 1% (see Figure S4).

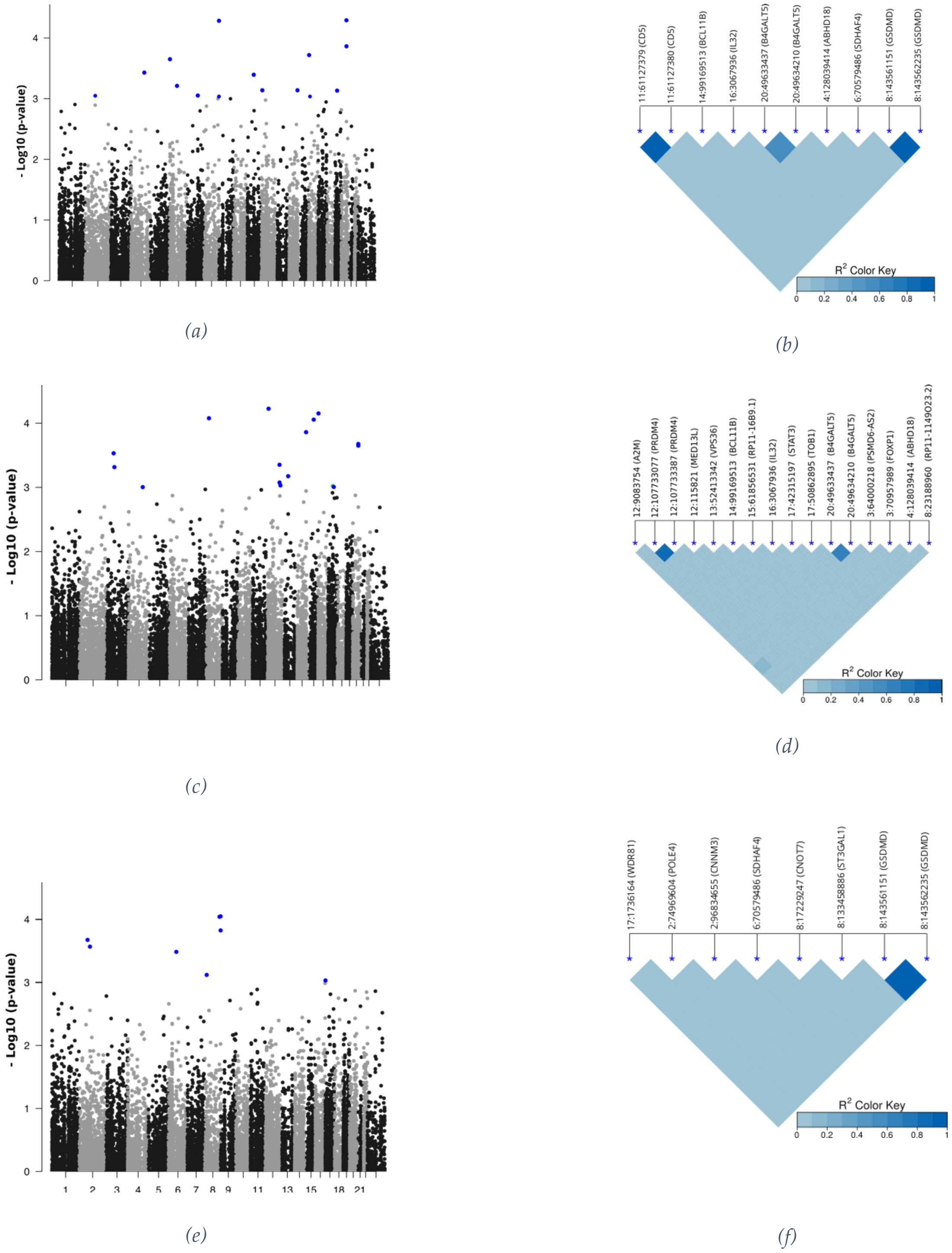

Figure S1_5. Manhattan plots to show the p-value of the evaluated variants (on the left side), and heatmap for linkag disequilibrium for the loci of the significant variants, which are identified in Table 2. (a) and (b) for cancer vs CP analysis. (c and (d) for rPDAC vs CP analysis. (e) and (f) for nrPC vs CP.

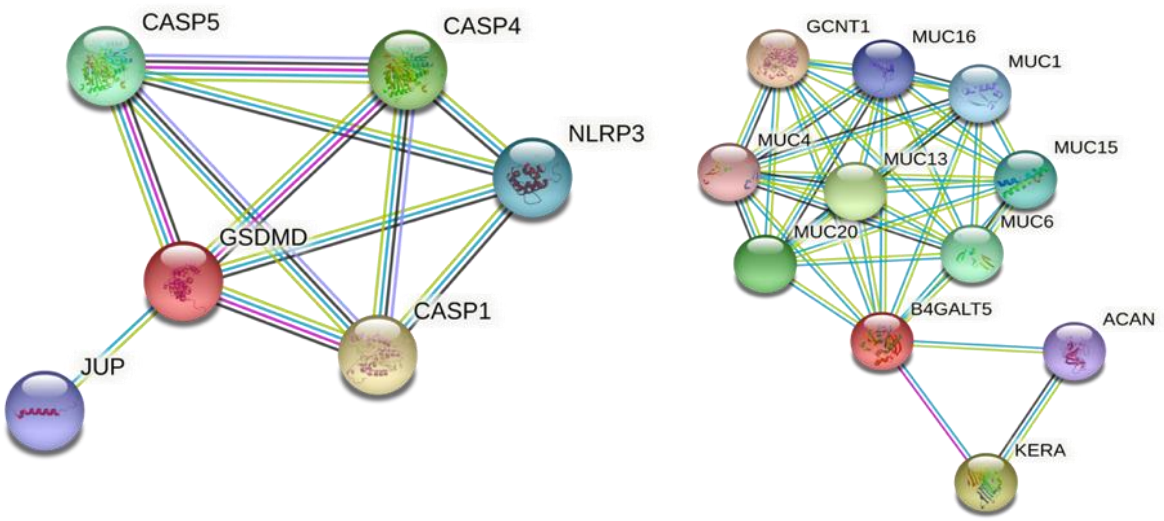

(a) *(b)*

Figure S1_6. Protein-protein interactions of the genes: B4GALT5 and GSDMD from STRING Database. The interactions include direct (physical) and indirect (functional) associations. (a) The Cluster of GSDMD shows its strong interactions with the caspase (CASP) gene family, which is related to the poor prognosis of pancreatic cancer. (b) Interactions of B4GALT5 show its associations with the MUC gene family that has a predefined role in diagnosis and early detection of pancreatic cancer.

Table S1_1: An overview of the functions and configurations for variants calling and quality control using GATK & PLINK.

**Function**	**Description and configuration**
1. Variants calling (HaplotypeCaller of GATK)	Call SNVs and INDELs simultaneously via local de novo assembly of haplotypes in the active region<br>--base-quality-score-threshold 18<br>--max-reads-per-alignment-start 50<br>--min-base-quality-score 10
2. Hierarchically merge (GATK)	Combine all GVCF files from each sample into one GVCF file.
3. Hard filters	SNVs & INDELs: --cluster-window-size 35<br>--cluster-size 3<br><br>For SNVs: --QD < 2.0. (QualByDepth)<br>Variant confidence normalized by unfiltered depth of variant samples (QD)<br>--MQ < 40.0<br>Root mean square of the mapping quality of reads across all samples (MQ)<br>--SOR > 3.0<br>Strand bias estimated by the symmetric odds ratio test (SOR)<br>--FS > 60.0<br>Strand bias estimated using Fisher's exact test (FS)<br>--MQRankSum < -12.5<br>Rank sum test for mapping qualities of REF versus ALT reads (MQRankSum)<br>--ReadPosRankSum < -8.0<br>Rank sum test for relative positioning of REF versus ALT alleles (ReadPosRankSum<br><br>--QD < 2.0<br>--SOR > 10<br>--FS > 200<br>--ReadPosRankSum < -20<br>For INDELs --InbreedingCoeff < -0.8
4. Check for missingness of varian	Filter variants with high missingness (higher than 3%), using PLINK.<br>--missing 0.03
5. Genotype quality (GQ) filter	Filter variant call bases which have low genotype quality score, using PLINK.<br>--GQ < 20
6. Check for minor allele frequency (MAF)	Filter variants with low minor allele frequency (MAF), using PLINK.<br>--maf 0.05
7. Check for Hardy-Weinberg-equilibrium	Filter variants are deviating from HWE P-value, using PLINK.<br>--hwe 1E-6
8. Check for missingness in sample	Filter samples with high missingness (10%), using PLINK.<br>--mind 0.1
9. Check for heterozygosity	Filter samples with too high or low heterozygosity rate, using PLINK.<br>heterozygosity > ± 3 SD (standard deviation)
10. Check for relatedness	Find pairs of samples looking too similar to each other by estimating the pairwise IBD, using PLINK.<br>PI_HAT > 0.2
11. Check for stratification	Check whether the samples belong to the same population by using complete linkage agglomerative clustering, based on pairwise identity-by-state (IBS) distance, and we detected outliers using PCA in PLINK.

Table S1_2. Performance of each fold in the cross-validation on training and test sets for deep learning model using variants and CA19-9 together.

Task	CA19-9		Fold	Performance on the training set Variants + CA19-9 clinical value					Performance on the test set Variants + CA19-9 clinical values				
	AUC	Acc.		AUC	Prec.	Rec.	F1	Acc.	AUC	Prec.	Rec.	F1	Acc.
**Cancer vs CP**	0.84	0.75	1	0.96	0.90	0.94	0.92	0.88	0.96	0.94	0.91	0.93	0.9
			2	0.93	0.90	0.92	0.91	0.88	0.96	0.90	0.90	0.90	0.90
			3	0.96	0.89	0.92	0.91	0.87	0.93	0.89	0.94	0.92	0.88
			4	0.95	0.89	0.92	0.90	0.86	0.95	0.85	0.97	0.91	0.87
			5	0.96	0.87	0.98	0.92	0.88	0.91	0.87	0.94	0.90	0.87
			**Average**	**0.95**	**0.89**	**0.94**	**0.91**	**0.87**	**0.94**	**0.89**	**0.93**	**0.91**	**0.88**
			**Ensemble**						**0.96**	**0.89**	**0.97**	**0.93**	**0.90**
**rPDAC vs CP**	0.84	0.76	1	0.99	0.91	0.98	0.95	0.94	0.91	0.93	0.88	0.90	0.90
			2	0.96	0.88	0.98	0.93	0.92	0.89	0.83	0.94	0.88	0.88
			3	0.99	0.96	0.98	0.97	0.97	0.93	0.79	0.94	0.86	0.85
			4	0.99	0.98	0.96	0.97	0.97	0.92	0.93	0.88	0.90	0.91
			5	0.99	0.93	0.98	0.95	0.95	0.90	0.93	0.88	0.90	0.91
			**Average**	**0.98**	**0.93**	**0.98**	**0.95**	**0.95**	**0.91**	**0.88**	**0.90**	**0.89**	**0.89**
			**Ensemble**						**0.96**	**0.93**	**0.88**	**0.90**	**0.91**
**nrPC vs CP**	0.84	0.76	1	0.9	0.76	0.88	0.81	0.79	0.85	0.78	0.95	0.86	0.83
			2	1.00	1.00	0.93	0.96	0.96	0.89	0.84	0.84	0.84	0.84
			3	0.98	0.89	1.00	0.94	0.94	0.83	0.75	0.95	0.84	0.81
			4	0.97	0.90	0.93	0.92	0.91	0.90	0.85	0.89	0.87	0.86
			5	0.83	0.95	0.67	0.79	0.81	0.82	0.93	0.68	0.79	0.81
			**Average**	**0.94**	**0.90**	**0.88**	**0.88**	**0.88**	**0.86**	**0.83**	**0.86**	**0.84**	**0.83**
			**Ensemble**						**0.92**	**0.75**	**0.95**	**0.84**	**0.81**

Table S1_3. Performance of each fold in the cross-validation on training and test sets for deep learning model using variants only.

Task	Fold	Performance on the training set Variants only					Performance on the test set Variants only				
		AUC	Prec.	Rec.	F1	Acc.	AUC	Prec.	Rec.	F1	Acc.
**Cancer vs CP**	1	0.86	0.89	0.84	0.8	0.81	0.84	0.81	0.83	0.82	0.75
	2	0.87	0.80	0.95	0.8	0.81	0.80	0.76	0.83	0.79	0.71
	3	0.84	0.82	0.89	0.8	0.79	0.81	0.80	0.80	0.80	0.73
	4	0.81	0.83	0.87	0.8	0.79	0.85	0.83	0.83	0.83	0.77
	5	0.90	0.85	0.93	0.8	0.84	0.79	0.78	0.80	0.79	0.71
	**Average**	**0.86**	**0.89**	**0.84**	**0.8**	**0.81**	**0.84**	**0.81**	**0.83**	**0.82**	**0.75**
	**Ensemble**						**0.83**	**0.76**	**0.83**	**0.79**	**0.71**
**rPDAC vs CP**	1	0.95	0.90	0.85	0.8	0.88	0.93	0.88	0.88	0.88	0.88
	2	0.99	0.96	0.94	0.9	0.95	0.86	0.93	0.81	0.87	0.88
	3	0.99	0.91	0.96	0.9	0.93	0.82	0.82	0.88	0.85	0.85
	4	0.97	0.85	0.98	0.9	0.90	0.88	0.83	0.94	0.88	0.88
	5	0.97	0.95	0.96	0.9	0.95	0.88	0.80	0.75	0.77	0.79
	**Average**	**0.97**	**0.91**	**0.94**	**0.9**	**0.92**	**0.87**	**0.85**	**0.85**	**0.85**	**0.86**
	**Ensemble**						**0.89**	**0.88**	**0.88**	**0.88**	**0.88**
**nrPC vs CP**	1	0.90	0.98	0.81	0.8	0.89	0.73	0.74	0.74	0.74	0.72
	2	0.91	0.60	1.00	0.7	0.65	0.82	0.6	1.00	0.75	0.64
	3	0.91	0.78	0.85	0.8	0.85	0.72	0.64	0.74	0.68	0.64
	4	0.74	0.64	0.74	0.6	0.64	0.74	0.64	0.74	0.68	0.64
	5	0.93	0.83	0.83	0.8	0.83	0.73	0.68	0.79	0.73	0.69
	**Average**	0.88	0.77	0.85	0.8	0.77	0.75	0.66	0.80	0.72	0.67
	**Ensemble**						**0.76**	**0.68**	**0.89**	**0.77**	**0.72**

Table S1_4. The significant predictors of the proportional hazard model for estimating the survival time of rPDAC patients. The hazard ratio (exponent of the regression coefficients) indicates the effect size of each variant. Hazard ratios well below one are good prognostic markers, and well above one are bad. For example, holding all other variants constant, the p-value for variant rs6728689 in SP100 was 6.87E-10, with a hazard ratio of around 14. This indicates a strong relationship to the bad prognostic (shorter survival time). The variant rs1362640215, however, with its hazard ratio equal to 0.05 and a p-value of 7.45E-04, was highly related to the good prognostic (longer survival time).

Marker Type	*Predictors*				*Regression Coefficients*	
	Chromosome	*Base Position*	*Rs Id*	*Gene*	*Hazard Ratio*	*p-Value*
Bad Prognostic	2	230545045	rs6728689	SP100	14.00	6.87E-10
	16	1964590	rs1141684	RPS2	9.43	9.63E-06
	1	205716224	rs951366	NUCKS1	7.55	2.96E-06
	2	68825777	.	ARHGAP25	5.59	2.29E-02
Good Prognostic	5	74627456	rs1362640215	ENC1	0.05	7.45E-04
	8	23107413	rs74766964	TNFRSF10C	0.09	6.59E-04
	4	57020984	rs2271806	POLR2B	0.10	3.43E-05
	1	225841073	rs4653694	TMEM63A	0.15	6.10E-04
	2	98600130	rs72823794	COA5	0.22	2.94E-05
	2	202303450	rs1054446	NOP58	0.23	3.18E-05
	16	30670495	rs11538957	FBRS	0.25	2.94E-02
	7	128028018	rs3808058	LRRC4	0.29	1.32E-02
	17	29551011	rs5819871	TAOK1	0.34	3.10E-03
	18	11884671	rs643652	GNAL	0.35	5.42E-03
	14	58271255	rs199843940	PSMA3	0.41	1.76E-02
	1	223766378	rs17599	CAPN2	0.43	1.70E-02

Table S1_5. Results of checking for proportionality assumption by using the Schoenfeld residuals against the transformed time. Having high p-values indicates that there are not time-dependent coefficients. The proportionality assumption is that the hazard rate of an individual is relatively constant in time. P-values for all of the selected predictors and the global hazard model are higher than 0.05.

Marker Type	*Predictors*				*p-Value*
	Chromosome	*Base Position*	*Rs Id*	*Gene*	
Bad Prognostic	2	230545045	rs6728689	SP100	0.43
	16	1964590	rs1141684	RPS2	0.30
	1	205716224	rs951366	NUCKS1	0.21
	2	68825777	.	ARHGAP25	0.89
Good Prognostic	5	74627456	rs1362640215	ENC1	0.25
	8	23107413	rs74766964	TNFRSF10C	0.80
	4	57020984	rs2271806	POLR2B	0.29
	1	225841073	rs4653694	TMEM63A	0.93
	2	98600130	rs72823794	COA5	0.86
	2	202303450	rs1054446	NOP58	0.64
	16	30670495	rs11538957	FBRS	0.51
	7	128028018	rs3808058	LRRC4	0.70
	17	29551011	rs5819871	TAOK1	0.33
	18	11884671	rs643652	GNAL	0.15
	14	58271255	rs199843940	PSMA3	0.17
	1	223766378	rs17599	CAPN2	0.42
Global model					0.48

Supplementary S2
‘ML-features.xlsx’
Information about the selected features for machine learning
